# Integrated metabolomics and network pharmacology to investigate the anti-hyperlipidemia effect of geniposidic acid on high-fat diet induced mice

**DOI:** 10.3389/fcell.2025.1655114

**Published:** 2025-09-08

**Authors:** Ruiting Tang, Kun Li, Mengting Liang, Pengwei Wang, Zeyun Li

**Affiliations:** ^1^ Department of Pharmacy, The First Affiliated Hospital of Zhengzhou University, Zhengzhou, Henan, China; ^2^ Henan Key Laboratory of Precision Clinical Pharmacy, Zhengzhou University, Zhengzhou, Henan, China; ^3^ Department of Pharmacy, Huanghe Science and Technology University, Zhengzhou, Henan, China; ^4^ Yichun University, Yichun, Jiangxi, China; ^5^ Department of Clinical Pharmacy, The First Affiliated Hospital of Xinxiang Medical University, Weihui, Henan, China

**Keywords:** metabolomics, network pharmacology, molecular docking, anti-hyperlipidemia, geniposidic acid

## Abstract

**Background:**

Geniposidic acid (GPA) has been reported to possess hypoglycemic, hypolipidemic, and choleretic properties. However, its efficacy against hyperlipidemia and the associated mechanisms remain inadequately defined.

**Methods:**

A hyperlipidemia model was established in mice using a high-fat diet, followed by a 12-week intervention with GPA or lovastatin (positive control). Serum biochemical parameters and Oil Red O staining were assessed to evaluate lipid-lowering effects. Furthermore, NMR- and MS-based metabolomics, network pharmacology, and molecular docking approaches were employed to explore the underlying mechanisms.

**Results:**

Biochemical analysis confirmed the lipid-lowering efficacy of GPA. Urinary metabolomics revealed that both GPA and lovastatin restored disturbed metabolic profiles, notably involving the TCA cycle, glycolysis, amino acid metabolism, and ketone body synthesis. Over 40 differential metabolites were identified, constructing a comprehensive metabolic network. Network pharmacology further enriched relevant metabolic pathways and screened key targets. Molecular docking demonstrated strong binding affinities between GPA and several core proteins, including ALB, CAT, ACACA, ACHE, and SOD1, suggesting these may be potential therapeutic targets.

**Conclusion:**

This study confirmed the anti-hyperlipidemic efficacy of GPA and, through integrated metabolomics and target prediction, elucidated its potential mechanisms of action. These findings provide a scientific basis for further research and offer a promising strategy for the development of novel antihyperlipidemic agents.

## Background

Hyperlipidemia, the most common type of dyslipidemia, has been well recognized as significantly associated with increased risks of cardiovascular disease, fatty liver, atherosclerosis, and acute pancreatitis (Nelson, 2012; Pirillo et al., 2021). Effective management of hyperlipidemia is an important strategy for precautions against these diseases. Synthetic medications are frequently reported to cause side effects such as diarrhea, nausea, myositis, and abnormal liver function, which hamper their application. In contrast, medicinal herbs have been reported to be beneficial for the management of hyperlipidemia (Rauf et al., 2022). Currently, plant-based therapies and natural products are regarded as important complementary medicines with full potential to improve hyperlipidemia.

Plant-based therapies, with thousands of years of history and minimal side effects, have attracted much interest and are becoming increasingly popular all over the world (Craig et al., 2021). Many plants, such as *Gardenia jasminoides* Ellis (Zhizi) and *Plantago asiatica* L. (Cheqian), have been used individually or in formulations to treat hyperlipidemia (Liu et al., 2012; Roghani-Shahraki et al., 2021). Natural products derived from such herbs, have become potential candidates for the development of new lipid-lowering drugs (Hasani-Ranjbar et al., 2010; Sabzghabaee et al., 2012). Various natural products have been obtained and have shown antihyperlipidemic effects in animal studies (El-Tantawy and Temraz, 2018). Geniposidic acid (GPA), an iridoid glucoside found in many herbs, including *G. jasminoides* and *P. asiatica*, has been proven to have cardiovascular, hypoglycemic, hypolipidemia, and choleretic activities (Akihisa et al., 2010; Hirata et al., 2011; Kim et al., 2013; Peng et al., 2017; Wang Y. et al., 2021; Cheng et al., 2022). Experimental studies have demonstrated that GPA effectively reduces lipid accumulation in HepG2 cells and *Caenorhabditis elegans*, and attenuates plaque formation in a rabbit model of atherosclerosis, thereby supporting its anti-hyperlipidemia potential (Huang et al., 2023; Gao et al., 2014). However, its anti-hyperlipidemia efficiency and remedial mechanisms are still not well defined.

Metabolomics, concerning the metabolic profiles of small-molecule metabolites, has been widely utilized in systems biology, including toxicological (Dinis-Oliveira, 2017), pharmaceutical (Puchades-Carrasco and Pineda-Lucena, 2015), and pathological studies (Dubin and Rhee, 2019). This could provide a holistic vision for pharmacodynamic evaluation and mechanistic studies of natural products (Yuliana et al., 2011; Halouska et al., 2011). As two main metabolomics platforms, a combination of Nuclear Magnetic Resonance (NMR) and high-resolution mass spectrometry (HRMS) techniques have been recognized as powerful methodologies for pharmacodynamic evaluation and mechanism research (Bingol and Brüschweiler, 2016).

Network pharmacology is a recently emerging systematic biology tool that generates complex interaction networks based on ligand compounds, potential target proteins, enriched pathways, and disease symptoms, thus shedding light on the pharmaceutical mechanisms underlying plant-based therapies (Wang X. et al., 2021). Molecular docking technology realizes virtual combination based on the three-dimensional structure of chemical analysis and the established protein target data, and evaluates the binding effect of chemical drugs or natural products with potential targets, which has become an important supplement to network pharmacology research (Trott and Olson, 2010). Network pharmacology, molecular docking integrated with metabolomic approaches provides a novel and holistic view to elaborate the mechanism of clinical applications or potential therapies of herbal medicine, as well as natural products (Sharma and Yadav, 2022).

In the present study, GPA and lovastatin (positive control) were orally administered to a hyperlipidemia mouse model established by high-fat diet (HFD) feeding. Together with conventional serum chemistry analysis, NMR combined with MS-based metabolomics, network pharmacology analyses and molecular docking were carried out to explore the anti-hyperlipidemic efficiency of GPA and to elucidate the underlying mechanisms.

## Materials and methods

### Chemicals and reagents

GPA (purity >98%) was obtained from Chengdu Biopurify Phytochemicals Ltd. (Chengdu, China). Lovastatin (purity >99%) was supplied by Dalian Meilun Biology Technology Co. Ltd. (Liaoning, China).

Acetonitrile (LC grade), formic acid (MS grade), and ammonium acetate (MS grade) were purchased from Fisher Scientific, Inc. (Newark, DE, United States). Deuterium oxide (99.9% D) and leucine enkephalin (for TOF-MASS calibration) were purchased from Sigma-Aldrich (St. Louis, MO, United States). Deionized water was prepared using a Milli-Q system (Millipore, Billerica, MA, United States). All other chemicals and reagents used were of analytical grade.

### Animal experiment

C57BL/6Slac mice (4 weeks, male, 10–13 g) were purchased from Shanghai SLAC Laboratory Animal Co. Ltd. (Shanghai, China) and kept in a humid (50%–60%), temperature (295–297 K), and light controlled (12 h light/dark cycle) environment, with *ad libitum* access to water and food. Animal experiments were performed in accordance with the National Institute of Health Guidelines regarding the principles of animal care (2020) and approved by the Institutional Animal Care and Use Committee, Huanghe Science and Technology College (No. 2022-0063).

All mice were acclimatized for 1 week and then randomly divided into five groups (*n* = 6) in different cages. Mice in the control group (Con) were fed with a low-fat control diet (D12450B, rodent diet with 10 Kcal% fat, Research Diets, Inc., New Brunswick, NJ, United States), while the other animals were fed with high-fat diet (D12492, rodent diet with 60 Kcal% fat, Research Diets, Inc., New Brunswick, NJ, United States) for 12 consecutive weeks to induce hyperlipidemia. During the induction period, HFD-fed mice were treated with lovastatin (30 mg/kg, Lov), GPA at low (100 mg/kg, GPA1), high (300 mg/kg, GPA2), or equal amounts of water (HFD). Mice in the control group received an equal amount of water. The intragastric administration lasted for 12 weeks (once daily). Pure water was used to dissolve the drugs.

At the end of the experiment, urine samples were collected overnight and centrifuged at 4 °C at 2,000 g for 10 min. Subsequently, serum samples were acquired via the arteria cruralis, followed by 1 h of standing and centrifugation at 4 °C at 2,000 g for 10 min. All samples were kept at −80 °C prior to use.

### Biochemistry assay

Serum biochemical assays were conducted using commercially available kits (Nanjing Jianchen Biotech Inc., Nanjing, China). Serum levels of total cholesterol (TC), triglyceride (TG), low-density lipoprotein cholesterol (LDL-C), and high-density lipoprotein cholesterol (HDL-C) were determined according to the manufacturer’s instructions. All samples were tested in duplicate.

### Oil Red O staining

Liver tissues were fixed in 4% paraformaldehyde solution at room temperature overnight. The fixed tissues were embedded in OCT, stored at −80 °C and sectioned. Frozen tissue sections were subjected to Oil Red O staining (Servicebio, China). After staining, images were scanned using a slide scanner (3DHISTECH Pannoramic SCAN, Hungary).

### ^1^H NMR and MS based metabolomics

For urine samples preparation and data acquisition, we followed the approach of our previous work (Li et al., 2015). A 1D NOESY (RD-90°-t1-90°-tm-90°-acquire) NMR pulse program with water suppression was employed for NMR data recording on a Bruker 600-MHz AVANCE III NMR spectrometer (Bruker, Germany). TOF MS Metabolomic profiles were acquired using the Waters ACQUITY™ UPLC-Q/Tof-MS system (Waters Co., Milford, MA, United States), with chromatograph separation achieved under a gradient elution program. Principal component analysis (PCA) and orthogonal partial least squares discriminant analysis (OPLS-DA) were employed to investigate differences among groups and identify significantly altered metabolites. NMR metabolites were assigned by comparison with standard compound spectra (https://www.hmdb.ca; https://www.bml-nmr.org) or screened against the Chenomx NMR software suite (Vers. 7.6, Chenomx, Inc., AB, CAN). Potential metabolic biomarkers revealed by MS were identified by searching the Human Metabolome Database (HMDB) and confirming MS2 fragmentation. The metabolic pathways involved were obtained from the Kyoto Encyclopedia of Genes and Genomes (KEGG) and the Human Metabolome Database (HMDB).

### Network pharmacology analysis

Putative molecular targets of GPA were obtained from the PharmMapper server (PharmMapper (lilab-ecust.cn)), and Prediction Target of Swiss Target Prediction network database (http://www.swisstargetprediction.ch/), as well as a prediction webserver for ATC codes and target prediction of compounds (https://prediction.charite.de/). The GeneCards (https://genecards.weizmann.ac.il/v3/) and OMIM (https://www.omim.org/) databases were used for target collections related to hyperlipidemia. All retrieved targets were uniformed in their official symbols by searching against UniProt Knowledgebase (http://www.uniprot.org/). The intersectional targets of molecular targets and hyperlipidemia-related targets were regarded as the predicted targets of GPA against hyperlipidemia. The gene and protein names of these targets were obtained from UniProtKB (http://www.uniprot.org/) for further analyses.

The ingredients-targets-pathways-disease network was constructed and visualized by Cytoscape 3.7.1 (http://cytoscape.org/). Pathway and Gene Ontology (GO) enrichment of the predicted targets was conducted using ClueGO in Cytoscape. KEGG pathway analysis was performed with a p-value of <0.05. The metabolites identified by metabolomics, together with the predicted targets collected, were imported into MetaboAnalyst (https://www.metaboanalyst.ca/) to obtain a compound-gene network to visualize the interactions among the metabolites and genes.

### Molecular docking

Molecular docking test was assessed using AutoDockTools V1.5.6 to confirm the interaction of GPA with proposed targets (Li et al., 2024). The top two core targets were evaluated to verify the method’s reliability, and receptor proteins were selected. The core protein crystal structures were obtained from the protein data bank (PDB, http://www.rcsb.org/), and the PyMol program was used to optimize the protein structures by deleting H_2_O. Hydrogens were inserted into the proteins, and the resulting charge was calculated with AutoDockTools V1.5.6 and exported as a PDBQT file. The molecular docking results were presented as docking scores; the higher the score, the greater the likelihood that a protein was a target of GPA.

### Statistics

All data are presented as the mean ± standard deviation (SD). GraphPad Prism 9.0 was used for statistical analysis between groups. Data results were identified using one-way analysis of variance (ANOVA). Statistical significance was set at *p* < 0.05.

## Results

### Biochemical and pathological analysis

**FIGURE 1:**
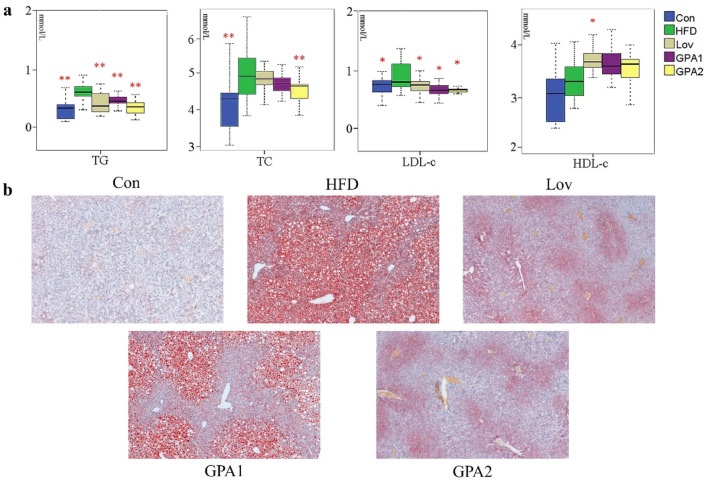
The effect of GPA on lipid accumulation in the liver of mice. **(a)** Boxplots for effects of GPA and lovastatin on serum TC, TG, LDL-C, HDL-C levels in hyperlipidemia mice (*n* = 6). The bottom of each box, the line drawn in the box and the top of the box represent the 1st, 2nd, and 3rd quartiles, respectively. The whiskers extend to ±1.5 times the interquartile range (from the 1st to 3rd quartile). **(b)** Oil red O staining of liver. Scar bar = 100 μm. *p < 0.05 and **p < 0.01 vs. HFD group.

To validate GPA’s antihyperlipidemic effect of GPA, serum levels of total cholesterol (TC), triglycerides (TG), high-density lipoprotein-cholesterol (HDL-C), and low-density lipoprotein-cholesterol (LDL-C) were tested. The levels of TC, TG, and LDL-C in the HFD group were remarkably higher than those in the control (Con) group (Figure 1), indicating successful induction of hyperlipidemia in mice. While after GPA or lovastatin treatment for 12 weeks, TG levels in GPA1, GPA2, and Lov groups were significantly reduced by 29.28%, 47.24%, and 34.17%; LDL-C levels were reduced by 27.88%, 28.62%, and 30.03% (P < 0.05), respectively. As for the TC levels, a significant reduction of 10.60% in GPA2 group was also noticed in contrast to the HFD group (P < 0.05). Meanwhile, HDL-C levels in Lov, GPA1, and GPA2 groups were all increased to some extent compared to the HFD group (11.88% for Lov, P < 0.05).

Oil red O staining was used to determine the accumulation of lipids in the liver of mice. The results showed that GPA reduced lipid accumulation in the liver of HFD mice in a dose-dependent manner, which was consistent with the results of biochemical analysis.

### ^1^H NMR based metabolomics analysis

**FIGURE 2:**
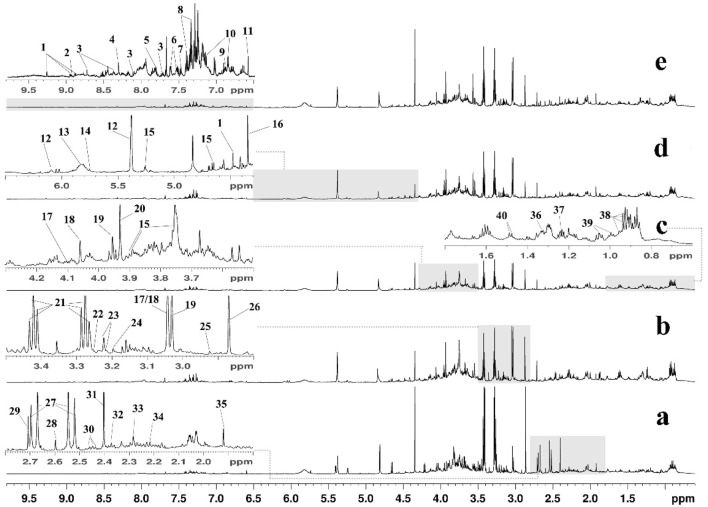
Typical 600 MHz ^1^H NMR spectra of mice urine from Con **(a)**, HFD **(b)**, Lov **(c)**, GPA1 **(d)** and GPA2 **(e)** groups. Metabolites: 1, 1-methylnicotinamide; 2, niacinamide; 3, nicotinamide N-oxide; 4, formate; 5, hippurate; 6, indole-3-acetate; 7, benzoate; 8, N-phenylacetylglycine; 9, tyrosine; 10, 4-hydroxyphenylacetate; 11, trans-aconitate; 12, allantoin; 13, urea; 14, cis-aconitate; 15, glucose; 16, tartrate; 17, cystine; 18, creatinine; 19, creatine phosphate; 20, creatine; 21, taurine; 22, betaine; 23, O-acetylcholine/O-phosphocholine; 24, choline; 25, N,N-dimethylglycine; 26, trimethylamine; 27, citrate; 28, methylamine; 29, dimethylamine; 30, glutamine; 31, succinate; 32, pyruvate; 33, acetoacetate; 34, acetone; 35, acetate; 36, lactate; 37, methylmalonate; 38, leucine/isoleucine; 39, valine; 40, alanine.

Typical ^1^H NMR spectra of urine samples from the Con, HFD, Lov, GPA1, and GPA2 groups are presented in Figure 2, with the major metabolites labeled. The identified metabolites, their assigned chemical shifts, and metabolic pathways are listed in Table 1. By comparing the spectra of the HFD group with those of the Con group, increased metabolites were identified as follows: creatinine, acetoacetate, and allantoin, and decreased metabolites were identified as taurine, trimethylamine (TMA), citrate, and succinate. After treatment with GPA, the acetate and taurine levels markedly increased. In addition, the spectra of the Lov and GPA groups were different, indicating differences in metabolic regulation. More detailed metabolic changes were analyzed using the orthogonal partial least squares discriminant analysis (OPLS-DA) model and are presented below.

**TABLE 1:** ^1^H Chemical shift assignment of the metabolites identified in urine and related metabolic pathways.

NO.	Metabolites	Moieties	δ1H(ppm)and multiplicity	Related pathway
1	1-methylnicotinamide		9.27(s); 8.96(d); 8.90(d); 8.16–8.20(t); 4.48(s)	Nicotinate and Nicotinamide Metabolism
2	Niacinamide	2-H,6-H	8.98(d); 8.70–8.71(dd);	Nicotinate and Nicotinamide Metabolism
3	Nicotinamide N-oxide	2-H,6-H,4-H,5-H	8.74(s); 8.47-8.49(d); 8.10–8.13(d); 7.12–7.14(m)	Nicotinate and Nicotinamide Metabolism
4	Formate	HCOO	8.46(s)	Folate Metabolism
5	Hippurate	Ph-H	7.81-7.84(d); 7.61–7.64(m); 7.53–7.56(t);	Gut microbiome-derived metabolism
6	Indole-3-acetate	Ph-H, NH	7.61–7.64(d); 7.49–7.51(d);	Tryptophan Metabolism
7	Benzoate	Ph-H	7.46–7.49(t)	Hippurate synthesis
8	N-phenylacetylglycine	Ph-H,NCH_2_CO	7.39–7.43(m); 7.33–7.37(m); 3.36–3.37(s)	Metabolites of fatty acids
9	Tyrosine	Ph-H	7.19–7.21(m); 6.89–6.94(d);	Tyrosine Metabolism
10	4-Hydroxyphenylacetat	Ph-H	7.14–7.16(d); 6.85–6.87(d);	Tyrosine Metabolism
11	trans-Aconitate	C=CH-CO	6.59–6.60(s)	
12	Allantoin	CONHCO, COCHN	6.09(s); 5.37–5.38(s)	Metabolites Related to oxidative stress and kidney damage
13	Urea	CO(NH_2_)_2_	5.7–6.0(s)	Urea Cycle
14	cis-Aconitate	C=CH-CO	5.74(t)	TCA cycle
15	glucose	CH	4.66(d)	Glycolysis and gluconeogenesis
16	Tartrate	COCOH-H	4.34–4.35(s)	
17	Cystine	NCHCO	4.09–4.12(m)	Taurine metabolism
18	Creatinine	N-CH_3_, N-CH_2_-	4.05(s); 3.04(s)	Creatine metabolism
19	Creatine phosphate	N-CH_3_, N-CH_2-_	3.94(s):3.04(s)	Creatine metabolism
20	Creatine	N-CH_3_, N-CH_2-_	3.92(s); 3.02(s)	Creatine metabolism
21	Taurine	N-CH_2_, S-CH_2_	3.25–3.30(t),3.40–3.44(t)	Bile acid biosynthesis and taurine metabolism
22	betaine	N-H_2_	3.25–3.26(s)	Choline metabolism
23	O-Acetylcholine/O-Phosphocholine	N(CH_3_)_3_	3.20–3.22(s)	Choline metabolism
24	Choline	N(CH_3_)_3_	3.18–3.19(s)	Choline metabolism
25	N,N- Dimethylglycine	N(CH_3_)_2_	2.92(s)	
26	Trimethylamine	N(CH_3_)_3_	2.86(s)	Gut microbiome-derived metabolism
27	Citrate	Half CH_2_, Half CH_2_	2.65–2.70(d),2.20–2.55(d)	TCA cycle
28	Methylamine	NCH_3_	2.59–2.60(s)	Gut microbiome-derived metabolism
29	Dimethylamine	N(CH_3_)_2_	2.70–2.71	Gut microbiome-derived metabolism
30	Glutamine	-NCO(CH_2_)_2_-	2.42–2.48(m),2.10–2.14(m)	Glutamate Metabolism
31	Succinate	(COCH_2_)_2_	2.40(s)	TCA cycle
32	Pyruvate	COCH_3_	2.37(s)	
33	Acetoacetate	COCH_3_	2.28(s)	Synthesis and degradation of ketone bodies
34	Acetone	CO(CH_3_)_2_	2.22(s)	Synthesis and degradation of ketone bodies
35	Acetate	COCH_3_	1.92(d)	
36	Lactate	βCH_3_	1.33(d)	Glycolysis and gluconeogenesis
37	Methylmalonate	CH_3_	1.23–1.25(d)	Propanoate Metabolism/Valine, Leucine and Isoleucine degradation
38	Leucine/isoleucine	δCH_3_	0.98(d)	Valine, leucine and isoleucine biosynthesis
		γCH_3_	0.94(d)	
39	Valine	γCH_3_	1.04(d); 0.98(d)	Valine, leucine and isoleucine biosynthesis
40	Alanine	βCH_3_	1.49–1.49(d)	Glycolysis and gluconeogenesis

Notes. s = singlet, d = doublet, dd = double doublet, t = triplet, q = quartet, m = multiplet.

**FIGURE 3:**
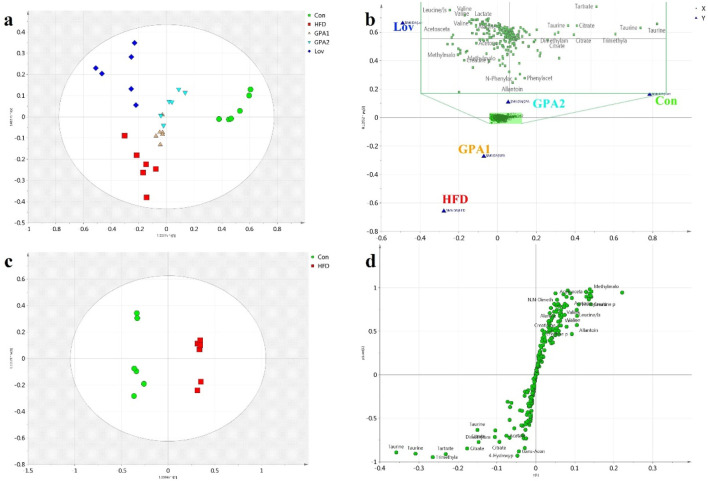
Metabolomics analysis by NMR platform. OPLS-DA score plot **(a)**, loading plot **(b)** of urine ^1^H NMR spectra obtained from Con, HFD, Lov, GPA1, and GPA2 groups; OPLS-DA score plot **(c)** and S-plot **(d)** of urine ^1^H NMR spectra obtained from Con and HFD groups.

First, urine samples from the five groups were analyzed using the OPLS-DA model. The score plot (Figure 3a) showed good separation among all groups (*R* ^*2*^ *Y* 0.932 and *Q* ^*2*^ 0.755). The dots in the HFD group were distributed far from those in the Con group, indicating a hyperlipidemic state. Meanwhile, the corresponding dots of the GPA or Lov groups deviated from those of the HFD group and approached those of the Con group, suggesting that GPA or lovastatin treatment effectively improved the disturbed hyperlipidemia metabolism profiles. Compared with Lov or GPA1 groups, GPA2 group was closer to the Con group, revealing a better therapeutic effect. These results indicated that GPA’s antihyperlipidemic effect was comparable to that of lovastatin. The variables with a significant influence on clustering are shown in Figure 3b. However, it was difficult to obtain deeper insight into the metabolic alterations in this plot. The OPLS-DA model between Con and HFD groups was conducted to detail the metabolic features of HFD induced hyperlipidemia. Obvious separation was observed (Figure 3c, *R* ^*2*^ *Y* = 0.994 and *Q* ^*2*^ = 0.962), confirming hyperlipidemia metabolic changes. The S-plot with significantly altered metabolites in the lower left or upper right quadrant shows the contributions of metabolites to discrimination (Figure 3D).

Similarly, to reveal the functional mechanisms of GPA and lovastatin, OPLS-DA analysis was conducted between the GPA2 and HFD groups, the Lov and HFD groups, and the GPA2 and Lov groups. The corresponding scores and S-plots are shown in Supplementary Figures S1–S3. Metabolites that were markedly different between the two groups were identified and discussed. Conclusions were drawn that GPA as well as lovastatin could significantly improve the metabolism of hyperlipidemic mice, and their regulatory mechanisms may be different.

**FIGURE 4:**
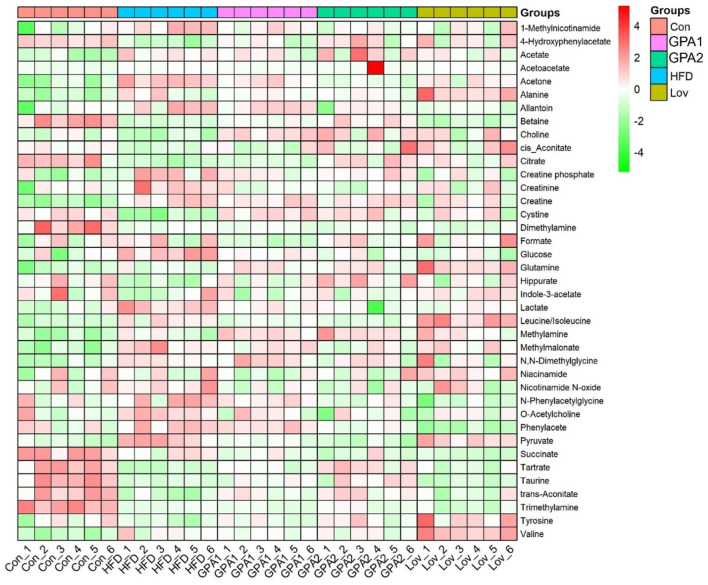
Heatmap of metabolites identified in NMR spectra.

In addition, the integral areas of the identified metabolites in the NMR spectra were displaced in a heatmap to visualize the metabolic characteristics of each group (Figure 4). Statistical analysis was conducted using one-way analysis of variance (ANOVA), followed by the least significant difference (LSD) *post hoc* test. A probability of *p* < 0.05 was regarded as statistically different among groups.

### MS based metabolomics analysis

**FIGURE 5:**
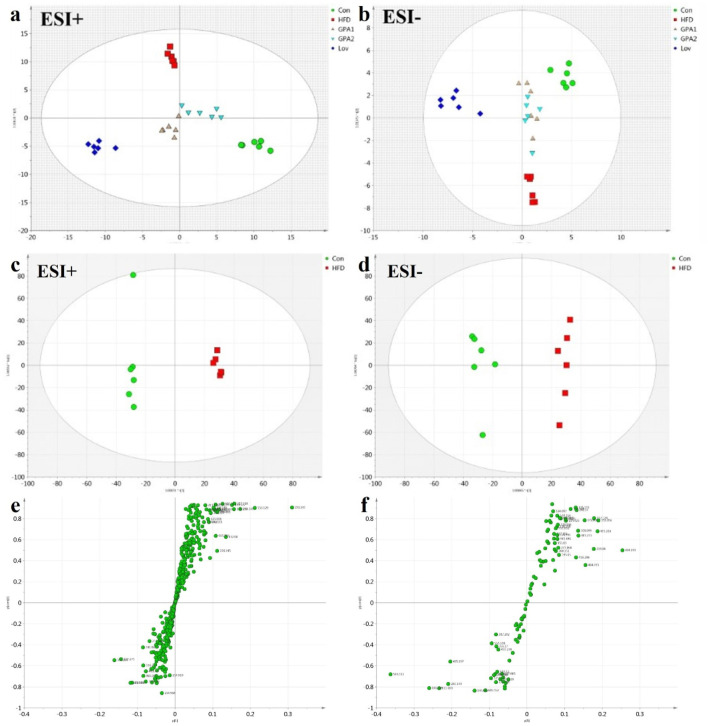
OPLS-DA analysis of UPLC-Q-TOF/MS data obtained in positive and negative ionization modes. **(a,b)** Score plots for Con, HFD, Lov, GPA1, and GPA2 groups in positive and negative modes. **(c,d)** Score plots for Con and HFD groups in positive and negative modes and according S-plots **(e,f)**.

**FIGURE 6:**
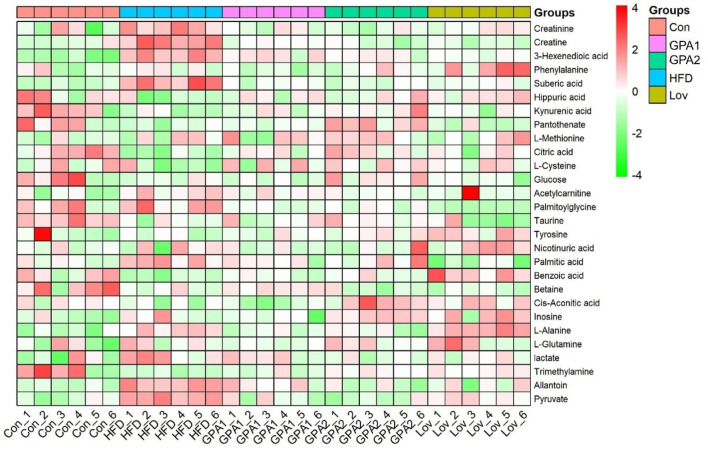
Heatmap of metabolites identified in MASS spectra.

The MS spectra of the urine samples were acquired in both ESI+ and ESI− modes. Representative base peak intensity (BPI) chromatograms are shown in Supplementary Figure S4. To reveal the antihyperlipidemic effects of GPA and lovastatin, OPLS-DA analysis of the model and treated groups was conducted. The score plots of OPLS-DA (Figures 5a,b) showed that the GPA- and lovastatin-treated groups clustered away from the HFD group in both ESI+ and ESI− modes, which was also observed in NMR analysis. In addition, to some extent, GPA and lovastatin recovered HFD-induced hyperlipidemia to normal control levels, confirming their anti-hyperlipidemic effects. To investigate endogenous metabolite alterations caused by HFD, OPLS-DA analysis was conducted between the hyperlipidemic and control groups (Figures 5c,d), suggesting that significant biochemical changes were induced by HFD feeding. The primary ions responsible for group discrimination are captured in the corresponding S-plot (Figures 5e,f). After screening with “VIP>1.00”, 78 (ESI+) and 12 (ESI-) metabolite variables were selected for further identification, of which 28 were identified. The identified metabolites, together with retention time, m/z, related pathways, are shown in Table 2. The variation among groups were displayed in Figure 6.

**TABLE 2:** Potential biomarkers identified by MS, together with retention time, measured molecular mass, related pathway.

HMDB ID	Metabolite	RT (min)	Observed m/z	Exact Mass	ESI mode	Adduct	Fragments	Related pathway
HMDB0000393	3-Hexenedioic acid	17.84	145.0332	144.0422	positive	M + H	127.0390,109.0284	β-Oxidation of fatty acids
HMDB0000210	Pantothenate	4.27	220.1176	219.1106	positive	M + H	185.21012	Pantothenate and CoA Biosynthesis
HMDB0000159	Phenylalanine	3.98	166.0738	165.0789	positive	M + H	119.9640, 13.9640	Phenylalanine metabolism
HMDB0000714	Hippuric acid	3.91	180.0847	179.0582	positive	M + H	105.0422,77.0012	Phenylalanine metabolism
HMDB0000715	Kynurenic acid	4.82	190.0510	189.0425	positive	M + H	143.9810	Tryptophan metabolism
HMDB0000893	Suberic acid	5.69	175.0811	174.0892	positive	M + H	158.0142, 99.9012	β-Oxidation of fatty acids
HMDB0000562	Creatinine	0.83	114.0676	113.0589	positive	M + H	86.00850	Creatine metabolism
HMDB0000064	Creatine	0.82	132.0799	131.0694	positive	M + H	90.2200, 44.0215	Creatine metabolism
HMDB0000094	Citric acid	0.86	191.0194	192.0270026	negative	M-H	111.0072,67.1089,87.0067	TCA circle
HMDB0000574	L-Cysteine	0.84	166.0174	121.0197	negative	M + FA-H	33.0212	Taurine metabolism
HMDB0000696	L-Methionine	5.07	150.0431	149.0510	positive	M + H	61.1350, 74.0902	Methionine metabolism
HMDB0000158	Tyrosine	1.81	182.0812	181.0738	positive	M + H	136.1240	Aromatic amino acid metabolism
HMDB0003269	Nicotinuric acid	4.96	181.0616	180.0535	positive	M + H	135.1244	Nicotinate and Nicotinamide Metabolism
HMDB0000220	Palmitic acid	17.86	255.2341	256.2402	negative	M-H		Metabolites of fatty acids
HMDB0000201	Acetylcarnitine	4.86	204.1350	203.1157	positive	M + H	84.0806	Energy metabolism pathways
HMDB0001870	Benzoic acid	5.22	121.0340	122.0367	negative	M-H	77.1007	Hippurate synthesis
HMDB0000043	Betaine	0.83	119.0674	118.0868	positive	M + H	59.0128	Choline metabolism
HMDB0000072	Cis-Aconitic acid	0.85	173.0090	174.0164	negative	M-H	84.98629	TCA cycle
HMDB0000122	D-Glucose	3.03	179.1010	180.06341	negative	M-H	89.0124, 118.9012	Glycolysis and gluconeogenesis
HMDB0000195	Inosine	1.97	267.0603	268.0807	negative	M-H	133.9956, 187.9406	Purine Metabolism
HMDB0000161	L-Alanine	0.94	90.0540	89.0476	positive	M + H		Glycolysis and gluconeogenesis
HMDB0000641	L-Glutamine	1.85	147.0746	146.0691	positive	M + H	83.9657	Glutamate Metabolism
HMDB0000190	L-Lactic acid	2.30	89.2772	90.0317	negative	M-H		Glycolysis and gluconeogenesis
HMDB0000906	Trimethylamine	14.6	76.0578	59.0735	positive	M + H		Gut microbiome-derived metabolism
HMDB0013034	Palmitoylglycine	17.7	314.2330	313.2616	positive	M + H	74.0251, 268.2520	Metabolites of fatty acids
HMDB0000462	Allantoin	4.31	159.0518	158.0439	positive	M + H	115.9576, 61.1640	Metabolites Related to oxidative stress and kidney damage
HMDB0000243	Pyruvate	0.85	87.1007	88.01604	negative	M-H		TCA cycle
HMDB0000251	Taurine	0.92	123.9102	125.0146	negative	M-H	106.8945, 79.8195	Taurine metabolism

### Network pharmacology study

**FIGURE 7:**
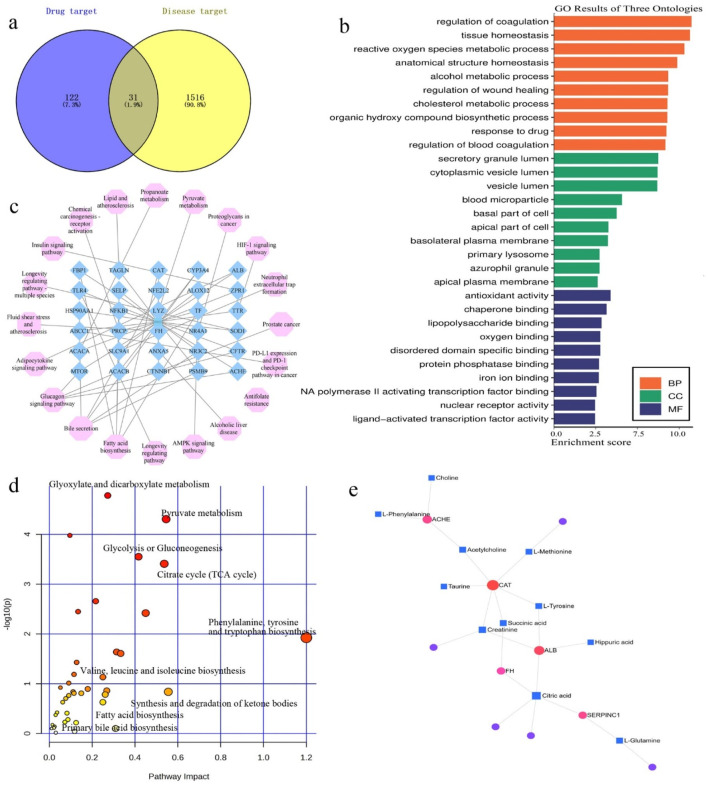
Network pharmacology analysis of GPA treating hyperlipidemia. **(a)** Venn diagrams of the predicated targets and hyperlipidemia retrieved targets; **(b)** The GO enrichment analysis of potential targets by ClueGO; **(c)** The KEGG pathways enrichment by 31 intersected targets. All pathways have a p-value of <0.05; **(d)** Joint metabolic pathways analysis by target and metabolites; **(e)** Target-metabolites network collected by Metabo-analyst.

For GPA molecular targets, 153 proteins were obtained from the Pharmapper, Swiss Target Prediction network, and SuperPred databases. For “hyperlipidemia-related targets,” 1555 proteins were retrieved from GeneCards and OMIM databases. The proteins were filtered using unions and duplicate values were removed. With the help of the Venny 2.1.0 tool (https://bioinfogp.cnb.csic.es/tools/venny/), 31 intersectional targets were obtained (Figure 7a). The STRING database was used to construct a protein-protein interaction (PPI) network. To elucidate the antihyperlipidemic effects of GPA, Gene Ontology (GO) and Kyoto Encyclopedia of Genes and Genomes (KEGG) pathway enrichment analyses were performed (Figure 7b). The main terms in the GO analysis were antioxidant activity (GO:0016209), reactive oxygen species metabolic process (GO:0072593), cholesterol metabolic process (GO:0008203), alcohol metabolic process (GO:0006066), and fatty acid biosynthetic process (GO:0006633). KEGG enrichment analysis revealed significantly affected pathways including the AMPK signaling pathway, alcoholic liver disease, pyruvate metabolism, lipid and atherosclerosis, insulin signaling pathway, adipocytokine signaling pathway, fatty acid biosynthesis, bile secretion, glucagon signaling pathway, and insulin resistance. The enriched joint metabolic pathways and component-target-disease network are illustrated in Figure 7c.

### Target screening and docking

To better understand the mechanisms underlying GPA’s antihyperlipidemic efficiency, an interaction network of identified metabolites and intersectional targets was established (Figure 7e). By checking the potential targets identified in the network pharmacology and the metabolites in the metabolic analysis, eight key targets were identified: ACACA, ACHE, ALB, CAT, FH, MTOR, SERPINC1 and SOD1. The metabolic pathways involved are associated with the TCA cycle, fatty acid biosynthesis, and oxidative stress (Figure 7d).

**FIGURE 8:**
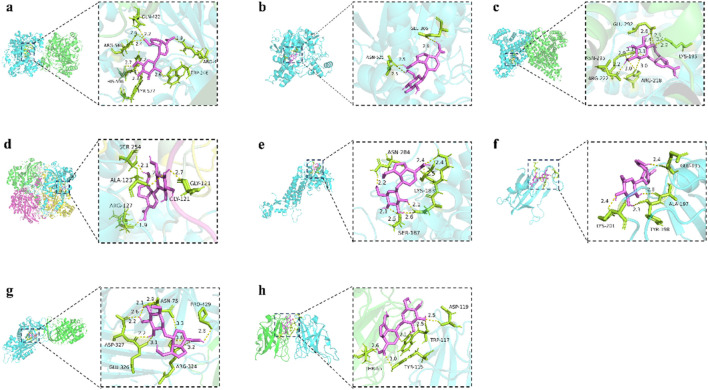
The protein ligands of the docking simulation. **(a)** Docking of GPA with ACACA. **(b)** Docking of GPA with ACHE. **(c)** Docking of GPA with ALB. **(d)** Docking of GPA with CAT. **(e)** Docking of GPA with FH. **(f)** Docking of GPA with MTOR. **(g)** Docking of GPA with SERPINC1. **(h)** Docking of GPA with SOD1.

Molecular-docking analysis was performed to confirm that eight key targets bound to GPA respectively. GPA was strongly bound to ALB, CAT, ACACA, ACHE, and SOD1, with binding energies were −7.7, −7.5, −7.3, −6.9, and −6.8 kJ/mol (Table 3), respectively. For GPA, Nine H-bonds were detected at Gln422, Arg546, His596, Tyr577, Trp246 and Arg478 on ACACA (Figure 8a). Three H-bonds were detected at ASN524 and GLU306 on ACHE (Figure 8b). Eleven H-bonds were detected at ASN295, ARG222, ARG218, LYS195 and GLU292 on ALB (Figure 8c). Four H-bonds were detected at ALA123, ARG127, GLY121 and SER254 on CAT (Figure 8d). Five H-bonds were detected at ASP119, TRP117, TYR115 and THR65 on SOD1 (Figure 8h). The binding energies between FH, MTOR, SERPINC1 and GPA are −6.3, −6.5 and −5.8 kJ/mol (Figures 8e–g), respectively, meaning lower binding activity to GPA than other active targets. The above indicates that GPA binds to the active site of the target.

**TABLE 3:** The binding energies of the target proteins to GPA.

Targets	Uniprot ID	Protein name	Binding energies (kJ·mol^-1^)
ALB	P02768	Albumin	−7.7
CAT	P04040	Catalase	−7.5
ACACA	Q13085	Acetyl-CoA carboxylase 1	−7.3
ACHE	P22303	Acetylcholinesterase	−6.9
SOD1	P00441	Superoxide dismutase [Cu-Zn]	−6.8
SERPINC1	P01008	Antithrombin-III	−6.5
FH	P07954	Fumarate hydratase, mitochondrial	−6.3
MTOR	P42345	Serine/threonine-protein kinase mTOR	−5.8

## Discussion

Hyperlipidemia is a metabolic disorder characterized by abnormally elevated levels of lipids (TG, TC, HDL-C and LDL-C) in the bloodstream (Wang et al., 2024). Its primary hazard lies in promoting atherosclerosis, thereby increasing the risk of cardiovascular diseases (Maulana and Ridwan, 2021). Existing first-line anti-hyperlipidemic drugs are limited in their clinical application due to varying degrees of adverse effects or efficacy constraints. For instance, statins are associated with musculoskeletal adverse reactions and impacts on blood glucose levels (Bell et al., 2024; Wang et al., 2015). Ezetimibe has limited lipid-lowering potency and poor patient adherence (Olmastroni et al., 2024; Rea et al., 2021). Additionally, PCSK9 inhibitors are hampered by their high cost (Xiang et al., 2023). Thus, there remains an urgent need to explore safe and effective anti-hyperlipidemic agents.

Plant-based therapies are fascinating and have attracted increasing attention. Natural products derived from related herbs are regarded as credible targets for drug development. As one of the most abundant components in GPA, one of the most abundant ingredients in *Plantaginis* semen (Ji-Ping et al., 2020) and *Gardeniae* Fructus (Wu et al., 2013), has been shown to have antihypertensive (Ishimitsu et al., 2021), anti-inflammatory (Tamura et al., 2022), anti-aging (Wang Y. et al., 2021), and neurotrophic effects (Zhou et al., 2019). Herbal medicines such as *Plantaginis* semen (Sun et al., 2019) and *Gardeniae* Fructus (Lee et al., 2005) have been reported to have lipid-regulating efficacy, leading to the speculation that GPA may also exert anti-hyperlipidemic activity. However, the antihyperlipidemic efficiency of GPA has not been explored, the underlying mechanism remains unknown. Therefore, investigating the anti-hyperlipidemic effect of GPA would play a positive role in the clinical development of novel anti-hyperlipidemic drugs. In this study, serum biochemistry, metabolomics, network pharmacology and molecular docking were integrated to address this efficiency.

Metabolomics has proven to be a powerful tool for elucidating the mechanisms underlying the efficacy of natural products. A combination of NMR and MS techniques provides a comprehensive platform for investigating metabolic characteristics. However, the combination proposed a requirement for the sample amount, which could not be guaranteed by serum samples from individual mice. Moreover, the limited number of serum samples was further reduced using biochemical analysis. Therefore, urine samples were used for metabolomic studies in this study. Previous studies have demonstrated that urinary metabolomics offers a reliable and non-invasive approach for investigating metabolic diseases (Zhao et al., 2021; Ji et al., 2018). In this study, due to the limited volume of mouse serum samples, urinary metabolomics was employed to overcome this constraint. This approach provided comprehensive metabolic regulation data and revealed regulatory networks, thereby contributing valuable insights into the pharmacological mechanisms of GPA against hyperlipidemia. Furthermore, network pharmacology and molecular docking were integrated as a supplementary tool to verify the findings of metabolomic analysis.

Serum biochemistry revealed that GPA significantly ameliorated the serum lipid profile of hyperlipidemia. Oil Red O staining of the liver also demonstrated that GPA dose-dependently reduced lipid deposition in the liver tissues of hyperlipidemic mice and alleviated hepatic steatosis, with the effect of the high-dose group being comparable to that of the positive control drug lovastatin. NMR and MS-based metabolomics studies showed that the urine metabolomics profile of HFD-induced mice drifted from that of the CON group, suggesting that biochemical changes occurred because of HFD feeding. Nevertheless, the GPA intervention-driven urine profiles of the treated groups regressed towards the Con group, verifying the hyperlipidemia-regulating effect of GPA. In addition, the GPA2 group showed better recovery from metabolic disorders caused by HFD feeding, suggesting a better anti-hyperlipidemic effect at a dose of 300 mg/kg body weight.

**FIGURE 9:**
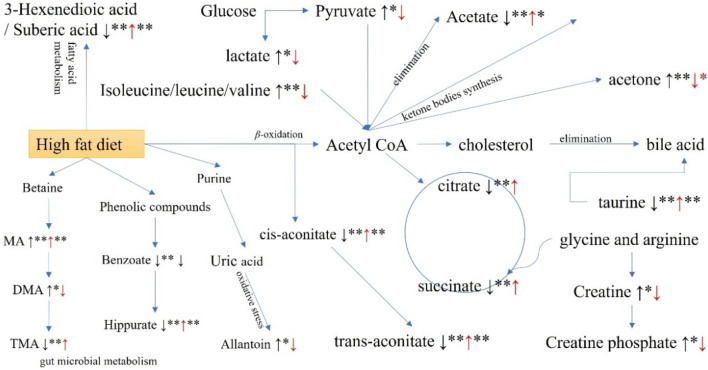
Potential metabolic pathways disturbed in hyperglycemia mice and regulated by GPA administration.

Based on the alterations in the identified biomarkers, a comprehensive metabolic sketch map of hyperlipidemia and GPA intervention was obtained (Figure 9). More than nine metabolic pathways were involved, including valine, leucine, and isoleucine biosynthesis; bile acid biosynthesis and taurine metabolism; glycolysis and gluconeogenesis; TCA cycle; *β*-oxidation of fatty acids; synthesis and degradation of ketone bodies; creatine metabolism; metabolites related to gut microbiota; metabolites related to oxidative stress; and the kynurenine pathway. Network pharmacology tentatively accounts for the mechanism by which GPA improves hyperlipidemia. According to KEGG pathway enrichment, pyruvate metabolism, lipid and atherosclerosis, insulin signaling pathway, fatty acid biosynthesis, bile secretion, glucagon signaling pathway, and insulin resistance were involved. GO term analysis revealed that antioxidant activity (GO:0016209), reactive oxygen species metabolic processes (GO:0072593), cholesterol metabolic processes (GO:0008203), and fatty acid biosynthetic processes (GO:0006633) were mediated by the antihyperlipidemic efficiency. After combining metabolomic findings with network pharmacology results, the selected metabolic pathways were expounded. Molecular docking verifies the binding activity between GPA and potential targets in the interaction network, further deepening the certainty of potential targets.

Isoleucine, leucine, and valine are ketogenic amino acids that can be quickly transformed into acetyl-CoA. The urine concentrations of isoleucine, leucine, and valine were significantly upregulated in the HFD group, which is considered to be related to insulin resistance (Newgard et al., 2009). Excessive acetyl-CoA generation was found to have a negative effect on the metabolism of ketogenic amino acids in HFD induced animals. Interestingly, reduced levels of valine, leucine, and isoleucine were also observed in the GPA2 group. This decrease was considered to indicate amelioration of insulin resistance by GPA treatment. Correspondingly, the insulin signaling pathway was revealed to be involved in the network pharmacology of GPA against hyperlipidemia.

Meanwhile, excessive acetyl-CoA promotes cholesterol and bile acid synthesis in hyperlipidemic mice. Taurine can be conjugated with bile acids to form bile salts, which is an important mechanism for the disposal of excess cholesterol. Herein, taurine levels were observably decreased in the urine of the HFD group, suggesting that the system attempted to eliminate excess cholesterol by converting it into bile salts (Murakami et al., 2000). In addition, taurine is an important antioxidant, and its decline may lead to oxidative stress in hyperlipidemia (Jiang et al., 2012). After GPA treatment, the taurine level was significantly increased, which may indicate a decline in the synthesis of cholesterol or bile acid or an improvement in oxidative stress. In line with the network pharmacology analysis, the pathways of bile secretion, antioxidant activity, and reactive oxygen species metabolic processes were also revealed to be involved in GPA’s antihyperlipidemic effect.

Pyruvate can enter the tricarboxylic acid (TCA) cycle or be converted into alanine and lactate. Increased pyruvate levels have been detected in hyperlipidemia, which could be explained by the suppression of the pyruvate dehydrogenase complex (Song et al., 2013). In addition, the reduction of dissolved oxygen, along with hyperlipidemia, may downregulate the conversion of pyruvate to acetyl-CoA, resulting in high levels of pyruvate and lactate (Jiang et al., 2013). The current study revealed increased levels of pyruvate, lactate, alanine, certain glucogenic amino acids (isoleucine and valine), and glucose in HFD mice, implying that glucose aerobic oxidation was inhibited, while glycolysis and gluconeogenesis were upregulated under hyperlipidemia. GPS interventions decreased the urine levels of glucose, pyruvate, and lactate, suggesting the suppression of gluconeogenesis and glycolysis. Abnormal glucose metabolism disturbed by the HFD was recovered by GPA treatment to some extent. This could possibly explain the hypoglycemic effects of GPA-containing herbs (Chen et al., 2014). Coincidentally, pyruvate metabolism, glucagon signaling pathway, and insulin resistance pathways were found to be associated with GPA’s antihyperlipidemic effects by network pharmacology analysis.

The TCA cycle, also known as the citric acid cycle, is the main energy-producing source under aerobic conditions. Our metabolomic analysis revealed that the levels of TCA cycle intermediates, including citrate, succinate, and trans-aconitate, were decreased in HFD-induced animals, suggesting downregulation of the TCA cycle and attenuation of glucose oxidation in the liver. This alteration indicated that the energy consumption pattern switched to lipid oxidation under hyperlipidemia. Fortunately, the urinary levels of citrate, succinate, and cis-aconitate increased after GPA intervention, indicating an upregulated TCA cycle and enhanced energy consumption back to the TCA cycle. This confirmed that GPA treatment partially improved abnormal energy metabolism in hyperlipidemia. This tendency was also revealed by network pharmacology analysis, as the glucagon signaling pathway is involved.

Acetate was significantly elevated in the case of hyperlipidemia due to the suppression of the TCA cycle and the dominating energy provision of lipid oxidation. Increased acetate levels have been reported in the urine of hyperlipidemic patients (Yang et al., 2019). Increased excretion of 3-hexenedioic acid and suberic acid was identified biomarkers of fatty acid metabolism disorder (Wang et al., 2019; Hagen et al., 1999). Consistently, these two dicarboxylic acids were increased in our study, indicating disorders of *β*-oxidation. However, the urine acetate level increased after GPA intervention, indicating improved elimination of *the β*-oxidation end-product in the form of urine acetate. As for 3-hexenedioic acid and suberic acid, decreased urine levels indicate an improvement in fatty acid *β*-oxidation. The involvement of fatty acid biosynthesis was also revealed using network pharmacology analysis.

An excess amount of acetyl-CoA is produced in hyperlipidemia, leading to enhanced synthesis of ketone bodies. Consistent with a previous report (Song et al., 2013), two important ketone bodies, acetoacetate and acetone, were increased in the urine samples of HFD induced mice in the present study. Nevertheless, urinary acetone and acetoacetate levels were subsequently decreased by GPA treatment, suggesting the suppression of ketone body synthesis and avoidance of ketoacidosis. This decrease was consistent with previous reports (Wang et al., 2013), implying enhanced acetyl-CoA elimination in other ways.

Creatine and creatine phosphate are well-recognized for their roles in energy metabolism. The urine levels of creatine and creatine phosphate have been reported to decrease in obesity or hyperlipidemia (Jiang et al., 2013). However, our metabolomic analysis showed an unexpected inconsistency, which may be explained by the differences in animal species and induction periods. Diet-mediated changes in gut microbiota have been reported during hyperlipidemia (Won et al., 2013). Dietary choline is broken into betaine, monoamine (MA), dimethylamine (DMA), and TMA by gut microflora (Asatoor and Simenhoff, 1965). Urine levels of choline metabolites are indicators of gut microbiota status (Piloquet et al., 2003). Hippurate, as microbial–host co-metabolite, inversely associated with obesity, has been identified as pivotal in mediating the beneficial metabolic improvements under Western-style diets (Nicholson et al., 2005; Lees et al., 2013). In line with previous studies (Jiang et al., 2013; Shearer et al., 2008), the urinary levels of choline metabolites and hippurate were significantly decreased by HFD in the current study. After GPA intervention, urine hippurate and benzoate levels increased, indicating partial recovery of the gut microbial metabolism. Meanwhile, the urine levels of TMA, DMA, and MA, which were disturbed by HFD feeding, also recovered to some extent.

Allantoin, a convenient biomarker of oxidative stress, was increased in the urine of the HFD group owing to the increased rate of superoxide production (Grootveld and Halliwell, 1987). Considering the decreased cysteine levels in the HFD group, the enhanced oxidative stress induced by HFD was verified. Urine allantoin levels declined significantly after the GPA intervention, indicating a reduction in oxidative stress. The remission of oxidative stress was also supported by network pharmacology analysis, with enriched GO terms of antioxidant activity (GO:0016209) and reactive oxygen species metabolic processes (GO:0072593).

According to serum biochemistry and metabolomic analyses, lovastatin treatment exhibits a significant antihyperlipidemic effect. However, metabolomic analysis revealed additional details. According to the OPLS-DA analysis between the Lov and HFD groups, the metabolic pathways disturbed by HFD induction were partially recovered. By the S-plot, the leucine and isoleucine levels were increased, indicating enhanced synthesis of ketogenesis amino acid by lovastatin; the acetate level were increased significantly, suggesting excretion of the excess amount of acetyl-CoA by *β*-oxidation by lovastatin; meanwhile, the citrate level was increased, inferring an upregulated TCA cycle; furthermore, urine levels of acetoacetate were increased, indicating enhanced ketone bodies synthesis under lovastatin treatment. However, when inspecting metabolic regulation of GPA (300 mg/kg), it was revealed that urine’s leucine and isoleucine levels were decreased, suggesting suppressed ketogenic amino acid synthesis; urine’s lactate levels were reduced, indicating attenuated glycolysis; urine’s citrate, trans-aconitate, and pyruvate levels were increased, indicating a recovered TCA circle; urine’s acetoacetate and acetate levels were reduced, indicating suppressed synthesis of ketone bodies. Based on the aforementioned metabolomic analysis, we can draw a tentative conclusion that the metabolic regulations of GPA and lovastatin were different.

ALB is the most abundant protein in blood, constituting approximately 60% of the total plasma proteins. In addition to maintaining the colloidal osmotic pressure of the blood, ALB also plays a key role in the transport of various substances, such as lipids and small-molecule drugs (Lu et al., 2008). Research has shown that hyperlipidemia, in combination with albuminuria and hypoalbuminemia, is a hallmark of nephrotic syndrome. The underlying mechanisms are complex, involving increased synthesis of lipoproteins in the liver and reduced clearance of lipoproteins from the circulation (Kaysen et al., 1987). Zhang Yuping et al. (Zhang et al., 2012) developed a centrifugal ultrafiltration-high-performance liquid chromatography (HPLC) method to screen and identify the binding of GPA to bovine serum albumin (BSA), thus confirming the interaction between GPA and ALB.

CAT and SOD1 are antioxidant enzymes. CAT catalyzes the decomposition of hydrogen peroxide (H2O2), produced by peroxisomal oxidases, into water and oxygen, thereby protecting cells from the toxic effects of hydrogen peroxide (Takeuchi et al., 1995). SOD1 is a key antioxidant enzyme that can neutralize radicals, which are typically generated within cells and are toxic to biological systems (Lin et al., 2013). It has been reported that GPA alleviates H_2_O_2_-induced oxidative stress in HACAT cells by activating the AKT/NRF2/OGG1 pathway (Cheng et al., 2022). Additionally, GPA enhances the enzymatic activity and gene expression levels of superoxide dismutase (SOD), while reducing the levels of reactive oxygen species (ROS) and malondialdehyde (MDA), thereby improving the survival rate of yeast under oxidative stress conditions (Wang Y. et al., 2021).

ACACA is a cytosolic enzyme that catalyzes the conversion of acetyl-CoA to malonyl-CoA, which is the first and rate-limiting step in *de novo* fatty acid synthesis (Kim et al., 2010; Colbert et al., 2010; Hunkeler et al., 2018). Increased ACACA activity promotes adipogenesis and fat accumulation, while GPA can inhibit lipid accumulation in HepG2 cells. Additionally, GPA can concentration-dependently reverse free fatty acid-induced triglyceride (TG) elevation, thereby exerting an anti-hyperlipidemic effect (Cheng et al., 2022). Therefore, GPA is likely to suppress the expression of ACACA, thereby inhibiting *de novo* fatty acid synthesis and reducing lipid accumulation.

ACHE is responsible for the rapid hydrolysis of the neurotransmitter acetylcholine released into the synaptic cleft, thus terminating neural signal transmission (Shafferman et al., 1992). It has been reported that plasma cholesterol levels are positively correlated with brain oxidative stress damage, leading to cognitive dysfunction (de Oliveira et al., 2011). This is because high cholesterol can significantly reduce ACHE activity, causing memory impairments in hyperlipidemic rats (Braun et al., 2017). Kaiser et al. (Kaizer et al., 2004) also demonstrated that in animals fed a high-saturated-fat diet for an extended period, ACHE activity and subsequent acetylcholine synthesis were inhibited in various brain regions. Given the potential interaction between GPA and ACHE, we hypothesize that GPA may not only improve hyperlipidemia but also restore ACHE activity reduced by high cholesterol, thereby enhancing cognitive function. The specific mechanism underlying the anti-hyperlipidemia effect of GPA warrants further experimental verification in future studies.

## Conclusion

In this study, a combined strategy integrating NMR- and TOF-MS-based urinary metabolomics with network pharmacology and molecular docking was employed to investigate the anti-hyperlipidemic effects of GPA. The results demonstrated that GPA ameliorates hyperlipidemia by modulating multiple metabolic pathways, particularly those related to amino acid metabolism, the TCA cycle, and ketone body synthesis. Notably, the metabolic pathways identified through metabolomics overlapped with those predicted by network pharmacology and molecular docking, supporting the reliability of the proposed mechanism. This multi-omics approach provides a comprehensive framework for elucidating the pharmacological actions of herbal compounds. This study lays the groundwork for understanding the anti-hyperlipidemic effects of GPA, which merits further investigation in future research.

## Data Availability

The original contributions presented in the study are included in the article/Supplementary Material, further inquiries can be directed to the corresponding author.

## Abbreviations

HFD, high fat diet; GPA, geniposidic acid; TCA, tricarboxylic acid; TG, triglycerides; TC, total cholesterol; HDL-c, high-density lipoprotein-cholesterol; LDL-c, low-density lipoprotein-cholesterol; PCA, principal component analysis; OPLS-DA, orthogonal partial least squares discriminant analysis; HMG-CoA, 3-hydroxy-3-methylglutaryl coenzyme A; NMR, nuclear magnetic resonance; MS, mass spectrometry; VIP, variable importance of projection; TMA, trimethylamine; DMA, dimethylamine; MA, methylamine.
