# Supplementary material for: Integrated metabolomics and network pharmacology to investigate the anti-hyperlipidemia effect of geniposidic acid on high-fat diet induced mice
Source: Front Cell Dev Biol. 2025 Sep 8;13:1655114. doi: 10.3389/fcell.2025.1655114 (PMC12450931; doi:10.3389/fcell.2025.1655114)
Supplement: Supplementary file 1 [file DataSheet1.docx]

**Supplemental Files**


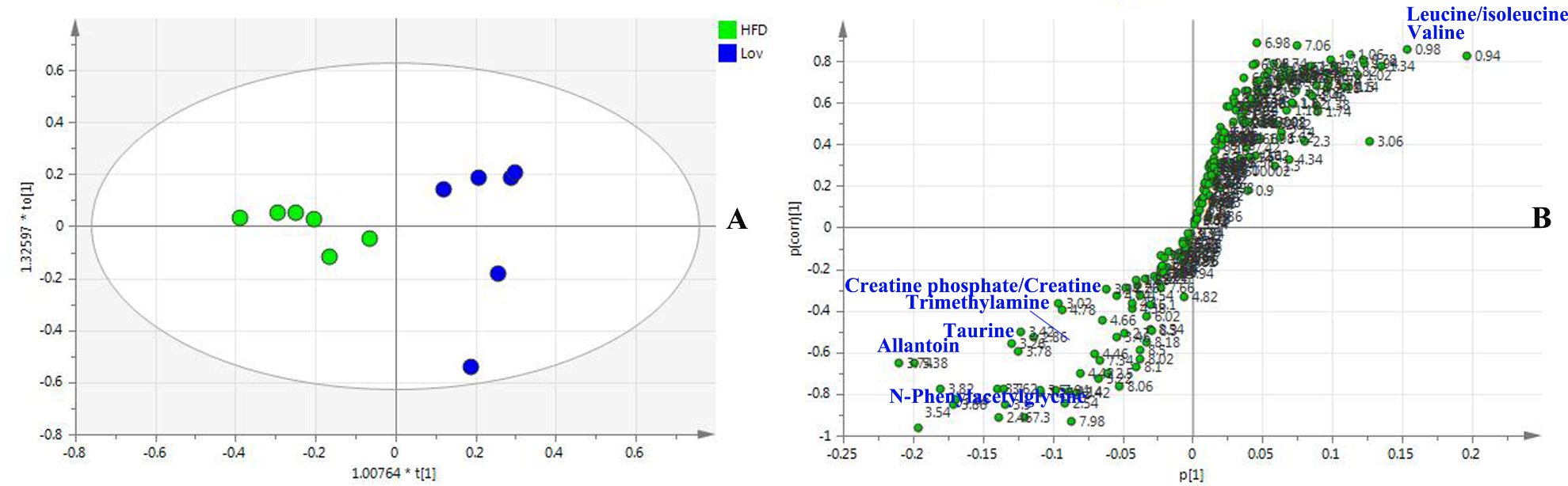


**Fig. S1** OPLS-DA score plot (a) and S-plot (b) of urine ^1^H NMR spectra obtained from Lov and HFD groups;


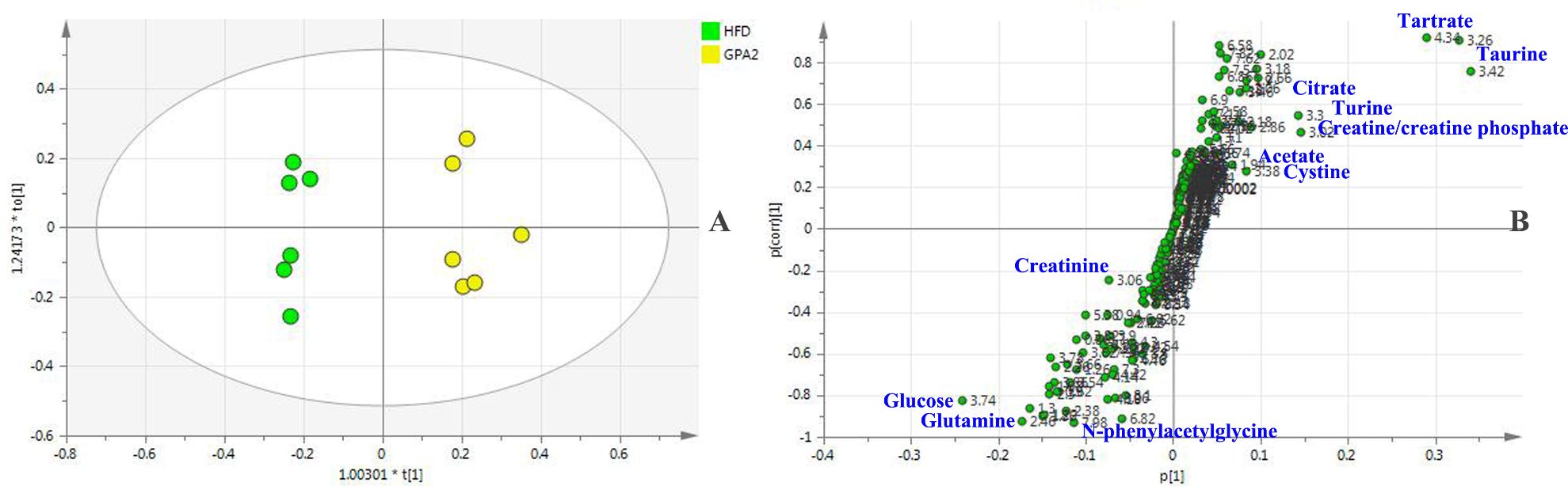


**Fig. S2** OPLS-DA score plot (a) and S-plot (b) of urine ^1^H NMR spectra obtained from GPA2 and HFD groups;


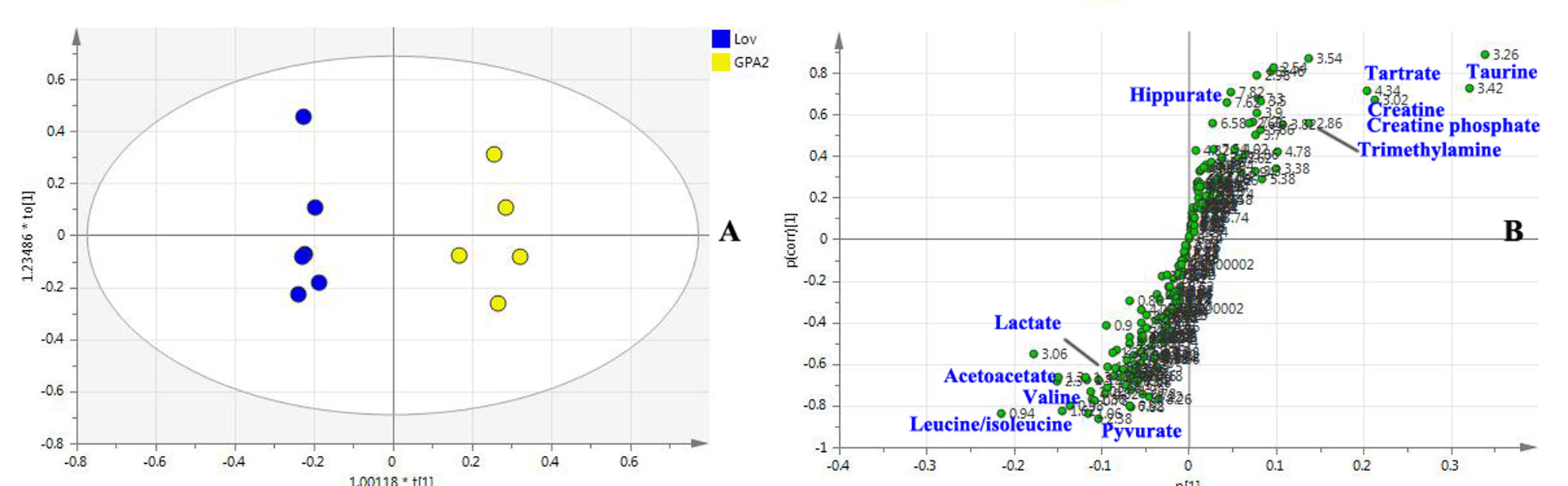


**Fig. S3** OPLS-DA score plot (a) and S-plot (b) of urine ^1^H NMR spectra obtained from GPA2 and Lov groups;





**Fig. S4** Representative base peak intensity chromatograms of urine samples from control, HFD, Lov, GPA2 groups in positive (a) and negative (b) ionization modes;
